# Sex differences in the relationships between body composition, fat distribution, and mitochondrial energy metabolism: a pilot study

**DOI:** 10.1186/s12986-022-00670-8

**Published:** 2022-05-21

**Authors:** Moriah P. Bellissimo, Candace C. Fleischer, David A. Reiter, Amy M. Goss, Lei Zhou, Matthew Ryan Smith, Jacob Kohlmeier, Rabindra Tirouvanziam, Phong H. Tran, Li Hao, Benjamin H. Crain, Greg D. Wells, Dean P. Jones, Thomas R. Ziegler, Jessica A. Alvarez

**Affiliations:** 1 Pauley Heart Center, Division of Cardiology, Department of Internal Medicine, Virginia Commonwealth University School of Medicine, Richmond, USA; 2 Division of Endocrinology, Metabolism and Lipids, Department of Medicine, Emory University School of Medicine, Atlanta, GA USA; 3 Emory Center for Clinical and Molecular Nutrition, Emory University, 101 Woodruff Circle NE, WMRB 1313, Atlanta, GA 30322 USA; 4 Department of Radiology and Imaging Sciences, Emory University School of Medicine, Atlanta, GA USA; 5 Department of Biomedical Engineering, Georgia Institute of Technology and Emory University, Atlanta, GA USA; 6 Department of Orthopedics, Emory University School of Medicine, Atlanta, GA USA; 7 Department of Nutrition Sciences, School of Health Professionals, University of Alabama at Birmingham, Birmingham, AL USA; 8 Center for Systems Imaging Core, Emory University School of Medicine, Atlanta, GA USA; 9 Division of Pulmonary, Allergy, Critical Care and Sleep Medicine, Department of Medicine, Emory University School of Medicine, Atlanta, GA USA; 10 Department of Microbiology and Immunology, Emory University School of Medicine, Atlanta, GA USA; 11 Division of Pulmonology, Allergy/Immunology, Cystic Fibrosis and Sleep/Apnea, Department of Pediatrics, Emory University School of Medicine, Atlanta, GA USA; 12 Translational Medicine, The Hospital for Sick Children, Toronto, Canada; 13 Atlanta Department of Veterans Affairs Medical Center, Decatur, GA USA

**Keywords:** Adiposity, Body composition, Fat distribution, Mitochondria, MRS, Muscle, Intermuscular fat

## Abstract

**Background:**

Adiposity and mitochondrial dysfunction are related factors contributing to metabolic disease development. This pilot study examined whether in vivo and ex vivo indices of mitochondrial metabolism were differentially associated with body composition in males and females.

**Methods:**

Thirty-four participants including 19 females (mean 27 yr) and 15 males (mean 29 yr) had body composition assessed by dual energy x-ray absorptiometry and magnetic resonance (MR) imaging. Monocyte reserve capacity and maximal oxygen consumption rate (OCR) were determined ex vivo using extracellular flux analysis. In vivo quadriceps mitochondrial function was measured using ^31^P-MR spectroscopy based on post-exercise recovery kinetics (τPCr). The homeostatic model assessment of insulin resistance (HOMA-IR) was calculated from fasting glucose and insulin levels. Variables were log-transformed, and Pearson correlations and partial correlations were used for analyses.

**Results:**

Mitochondrial metabolism was similar between sexes (*p* > 0.05). In males only, higher fat mass percent (FM%) was correlated with lower reserve capacity (r = − 0.73; *p* = 0.002) and reduced muscle mitochondrial function (r = 0.58, *p* = 0.02). Thigh subcutaneous adipose tissue was inversely related to reserve capacity in males (r = − 0.75, *p* = 0.001), but in females was correlated to higher maximal OCR (r = 0.48, *p* = 0.046), independent of FM. In females, lean mass was related to greater reserve capacity (r = 0.47, *p* = 0.04). In all participants, insulin (r = 0.35; *p* = 0.04) and HOMA-IR (r = 0.34; *p* = 0.05) were associated with a higher τPCr.

**Conclusions:**

These novel findings demonstrate distinct sex-dependent associations between monocyte and skeletal muscle mitochondrial metabolism with body composition. With further study, increased understanding of these relationships may inform sex-specific interventions to improve mitochondrial function and metabolic health.

**Supplementary Information:**

The online version contains supplementary material available at 10.1186/s12986-022-00670-8.

## Introduction

Excess adiposity causes metabolic perturbations and increased metabolic disease risk [1]. While body mass index (BMI) is strongly correlated with mortality risk, BMI lacks the sensitivity to determine underpinnings of adiposity and disease risk at an individual level [2]. Rather, assessment of body composition, including fat distribution, conveys information specific to components of body weight (lean, fat, and bone mass), fat partitioning patterns, and how these factors uniquely influence disease risk. Both epidemiologic and clinical evidence demonstrate that location of fat deposition impacts an individual’s health and disease risk. For example, accumulation of fat in the abdominal area, a predominant fat pattern in males, is closely linked to insulin resistance and cardiometabolic disease [3]. In contrast, fat stored in the gluteal-femoral region, a fat pattern predominantly observed in females, is associated with protection against metabolic diseases [4]. The underlying mechanisms linking these sexual dimorphisms of fat storage location to differences in disease risk is largely unexplored.

Mitochondrial activity and function may influence sex differences in disease occurrence [5, 6]. Mitochondria are integral for both cellular energy metabolism and disease progression, and research suggests that mitochondrial processes differ in males and females [5]. Diseases linked to impaired mitochondrial metabolism and decreased energy production include cardiometabolic and neurodegenerative diseases, as well as diseases of aging [5, 7–9]. Mitochondrial dysfunction promotes disease processes through increased production of oxidant species, fostering a pro-inflammatory cellular environment, and results in macromolecular damage [10]. Oxidative phosphorylation serves as a fundamental measure of mitochondrial energy metabolism and can be assessed using in vivo and ex vivo techniques [11–13]. Monitoring mitochondrial function could be an early indicator of disease before classic clinical symptoms are detected.

Alterations in mitochondrial function linked to excess adiposity have been documented [10, 14]; however, few studies have investigated the role of body composition and fat distribution with mitochondrial metabolism [15, 16]. Also, while lower body fat deposition patterns are linked to reduced disease risk [4], no study has investigated if fat location is related to mitochondrial metabolism. Most studies only investigate a single tissue assessment of mitochondrial respiration related to disease. Additionally, there is a lack of research investigating whether body composition is differentially related to mitochondrial function by sex, and if fat distribution is related to mitochondrial function to a greater extent than whole body adiposity [15–21].

One of the challenges in nutritional research is the collection of relevant tissues to assess mitochondrial function in living subjects, as tissue biopsies are often highly invasive. However, recent advances in assessing mitochondrial function in platelets and circulating peripheral blood mononuclear cells have shown promise as metabolic biosensors to assess systemic mitochondrial perturbations [21, 22]. This pilot study sought to determine the inter-relationships  between body composition and fat distribution and in vivo and ex vivo indices of mitochondrial oxidative phosphorylation (energy metabolism) in a cohort of adults and whether these relationships differed by sex. To provide a clinically relevant context, a secondary aim was to test for associations between mitochondrial metabolism and clinical biomarkers.

## Methods

### Population and study design

This pilot, cross-sectional study included 34 healthy adults with no reported illnesses. Adults with a self-reported BMI within the normal weight, overweight, or obese BMI categories were recruited using flyers and by word of mouth. To be enrolled, participants had to be 18 years of age or older, have independent ambulatory status, and no hospitalization within the previous year. Adults were excluded from the study if they reported a diagnosis of a chronic metabolic, infectious, respiratory, malignant (other than localized basal cell skin cancer), pro-inflammatory, or auto-immune disease; BMI ≥ 35; medications affecting body composition; > 10% body weight loss in the last six months; currently pregnant or lactating; a limb amputation; permanent metal implants or internal metal; muscle pain preventing participation in study procedures; or a history of drug or alcohol abuse. The study was approved by the Emory University Institutional Review Board (IRB), and all participants read and signed an IRB-approved consent form prior to enrollment. Following an overnight fast (≥ 10 h), subjects reported to the Emory University Hospital Clinical Research Unit. Fasting was confirmed prior to initiating study procedures.

### Body composition and fat distribution

A total body scan was conducted on all subjects using dual energy x-ray absorptiometry (DXA, GE Lunar iDXA, GE Healthcare, Madison, WI, USA), and visceral adipose tissue (VAT) was measured using CoreScan™ software. Height and weight were measured without shoes using a manual stadiometer and electronic scale, respectively.

Mid-thigh tissue area was assessed from an axial T1-weighted magnetic resonance (MR) image collected using a 3.0-Tesla (3 T) Siemens MAGNETOM PrismaFIT whole body magnetic resonance imaging (MRI) scanner (Siemens Medical Solutions, Erlangen, Germany). The center image used for thigh composition analysis (3 mm thickness) was acquired from the midpoint of the patella and hip. All scans were analyzed individually using sliceOmatic image analysis software (version 5.0, TomoVision, Quebec, Canada) to obtain thigh subcutaneous adipose tissue (thigh SAT), intermuscular adipose tissue (thigh IMAT), and total muscle area [23].

### Ex vivo monocyte mitochondrial function

Fasted peripheral blood draws were performed, and 12 mL whole blood was collected in EDTA tubes for isolation of monocytes following treatment with a RosetteSep™ Human Monocyte Enrichment Cocktail (STEMCELL Technologies Canada Inc, Vancouver, British Columbia, Canada). An 8-well Seahorse Bioscience XFp extracellular flux analyzer (Agilent Technologies, Santa Clara, CA) was used for real-time measurement of mitochondrial respiration. Each 8-well microplate was prepared the day prior to testing, and each well was coated with Cell-Tak. Isolated monocytes were plated at 200,000 cells/well in XF Base Medium with 5.5 mM glucose, 1 mM pyruvate, and 4 mM glutamine. Monocytes from each individual participant were plated in triplicate (n = 3 technical replicates per individual). After seeding cells, the microplates were centrifuged at 300 g for 1 min. Prior to testing, all sensor cartridges passed oxygen consumption rate (OCR, pmol/min) quality control tests. The Mito Stress Test Kit (Agilent Technologies, Inc., Santa Clara, CA), which applies a sequence of inhibitors and uncouplers in oxidative phosphorylation, was used to assess cellular respiration [13]. Evaluation of mitochondrial respiration (mitochondrial function) included initial measurement of basal OCR, followed sequentially by the addition of oligomycin (2.0 µM, an ATP synthase inhibitor), carbonyl cyanide-4 (trifluoromethoxy) phenylhydrazone (FCCP, 0.5 µM, an uncoupling agent), and lastly by rotenone and antimycin A (0.5 µM, complex I and complex III inhibitors, respectively). Data from replicate wells per subject were averaged. Maximal OCR was assessed after the addition of FCCP, and reserve capacity was calculated as the difference between maximal OCR and basal OCR and applied as a measure of the cells’ ability to respond to an increase in energy demand, with higher values indicating better mitochondrial function.

### In vivo skeletal muscle mitochondrial function

In vivo measurements of phosphorus-containing metabolites were acquired using ^31^P-magnetic resonance spectroscopy (^31^P-MRS) of the quadriceps muscles. Within 1 h prior to the ^31^P-MRS measurement, each subject was provided a meal (Additional file 1: Table S1) and practiced the standardized exercise (described below) on their dominant leg outside of the MR scanner. All MR measurements were performed on a Siemens MAGNETOM PrismaFIT 3 T whole body MR scanner (Siemens Medical Solutions, Erlangen, Germany). Participants were positioned in the scanner bore feet first and supine with a leg rest pillow under their knees (Newmatic Medical, Caledonia, MI). A dual-tuned hydrogen-phosphorous (^1^H-^31^P) flexible surface coil (RAPID MR International, LLC, Columbus, OH) was tightly secured to the participants' non-dominant leg at the midpoint of their quadriceps. The participants’ thighs and hips were secured on the bed of the MR scanner with straps to focus the exercise to the quadriceps muscles. In the MR scanner, participants listened to a recording and knee extension exercises were performed when prompted by a metronome for one minute while wearing non-magnetic ankle weights corresponding to 12% of their DXA-measured lean mass. MRS was acquired continuously from the thigh during rest (1 min), exercise (1 min), and recovery (8 min) using a non-localized free induction decay sequence (TR = 1 s; TE = 0.22 ms; 6 averages; 90º flip angle; 1024 complex data points; 2000 Hz bandwidth) and adiabatic half passage (AHP) excitation pulses. Prior to acquisition of dynamic MRS, a fully relaxed, unsaturated spectrum was acquired with the same parameters except with TR = 15 s. Spectra were analyzed with jMRUI version 5.2 [24] using the advanced method for accurate, robust, and efficient spectral fitting (AMARES) [25] in the time domain after applying a 5 Hz Lorenztian apodization filter. The phosphocreatine (PCr) recovery time constant (τ) was calculated from spectra acquired during the time-period after the cessation of exercise with a monoexponential fit using MATLAB (R2017b, Mathworks, Natick MA). This time constant represents the inverse of in vivo maximum oxidative ATP synthesis, where smaller values of τPCr reflect greater ATP synthesis (i.e., a shorter time to recovery). Initial PCr concentration at rest was estimated from the fully relaxed ^31^P-MR spectrum by normalizing the PCr peak area by the total γ-ATP peak area, under the assumption that ATP concentration is 5.5 mmol/kg wet weight at rest [26]. ATP maximal production rate (ATPmax) was calculated as the initial concentration of PCr at rest divided by τPCr [27], where higher values indicate higher rates of ATP production.

### Clinical measures

Fasting serum glucose and insulin were measured in the University of Alabama at Birmingham Metabolism Core. Serum glucose levels were assessed using a glucose oxidase assay on a Stanbio Sirus automated analyzer (Stanbio Laboratory, Boerne, TX, USA). Serum insulin was measured by immunofluorescence on a TOSOH AIA-II analyzer (TOSOH Corp., South San Francisco, CA, USA; intra-assay CV of 1.5% and interassay CV of 4.4%). The homeostatic model assessment of insulin resistance (HOMA-IR) was calculated as glucose mg/dL x insulin Uu/mL ÷ 405 [28].

### Physical activity

All participants reported physical activity levels by completing the long form of the international physical activity questionnaire (IPAQ). Collected data was summed by domain and intensity, then converted to MET-minutes per week for walking (MET 3.3), moderate-intensity (MET 4.0), and vigorous-intensity activity (MET 8.0). MET values utilized were based on average MET values for activity intensities and the IPAQ Reliability Study [29]. Participants were categorized as having high, moderate, or low levels of activity according to the guidelines for analyzing IPAQ data [29].

### Statistical analyses

Descriptive statistics for all variables are presented as mean ± standard deviation or counts and proportions for categorical variables. A Fisher’s exact test was used to test for differences in race distribution by sex, and a Student’s t-tests was used to investigate differences in age by sex. Mitochondrial energy metabolism variables and body composition variables were log transformed for analyses and back transformed for data presentation as geometric mean and 95% confidence interval. Prior to log transformation, median group values were imputed for two subjects with missing data for monocyte mitochondrial respiration. Student’s T-tests were used to examine differences in mitochondrial metabolism and body composition parameters between males and females. To test if sex moderated the relationships between body composition, fat distribution, and mitochondrial function, separate linear models were used with mitochondrial function as the outcome and a product term between sex and the body composition variable of interest. A similar approach was used to assess an interaction with race. Pearson correlations were used to test for associations between body composition and skeletal muscle (τPCr, ATPmax) and monocyte mitochondrial metabolism (maximal OCR, reserve capacity) measures. Partial Pearson correlations determined associations between VAT and thigh SAT after controlling for fat mass with skeletal muscle and monocyte mitochondrial metabolism variables. Partial Pearson correlations were also used to evaluate associations between thigh IMAT and mitochondrial respiration variables, additionally controlling for thigh muscle area. To address the secondary aim, Pearson correlations were used to examine relationships between clinical biomarkers with measures of mitochondrial metabolism. All statistical analyses were performed in JMP Pro software version 15 (SAS Institute Inc, Cary, NC, USA).

## Results

Demographic and clinical characteristics for all participants (N = 34) and by sex are presented in Table 1. The study cohort was predominantly comprised of Caucasians, with 19 females and 15 males. The mean age was 27.9 years (range 18–36). There was a significant difference in distribution of race between males and females (*p* = 0.03), and age (*p* = 0.18) and BMI (*p* = 0.24) were similar between males and females. Fasting serum glucose, insulin, and HOMA-IR levels were similar between males and females (*p* > 0.05), and average values were within normal ranges. There were no differences between males and females for self-reported levels of physical activity (*p* = 0.97), and most participants reported high (47%) or moderate (41%) activity levels.

**Table 1:** Demographic characteristics of the cohort

	All Subjects N = 34	Females n = 19	Males n = 15	*p* value
Race, n (%)				0.03
Caucasian	20 (59)	13 (68)	7 (47)	
Asian	11 (32)	3 (16)	8 (53)	
African American	3 (9)	3 (16)	–	
Age (y)	27.9 ± 5.0	26.8 ± 4.7	29.1 ± 5.0	0.18
BMI (kg/m^2^)	24.7 ± 4.0	25.6 ± 4.1	24.0 ± 3.8	0.24
Fasting serum glucose (mg/dL)	90.6 ± 8.0	88.6 ± 5.9	93.1 ± 9.6	0.11
Fasting serum insulin (Uu/mL)	6.0 ± 4.0	5.8 ± 3.3	6.4 ± 4.7	0.66
HOMA-IR	1.4 ± 1.0	1.3 ± 0.7	1.5 ± 1.3	0.47
Physical activity levels, n (%)				0.97
Low	4 (12)	2 (11)	2 (13)	
Moderate	14 (41)	8 (42)	6 (40)	
High	16 (47)	9 (47)	7 (47)	

Data are presented as n (%) or mean ± SD

BMI, body mass index; HOMA-IR, homeostatic model assessment of insulin resistance

Body composition measures are shown in Table 2. Females had higher fat mass percent and thigh fat compared to males, and males had higher lean mass and VAT. Descriptive variables from the ex vivo and in vivo assessments of oxidative phosphorylation are also shown in Table 2, with no differences found between males and females (*p* > 0.05). After adjusting for body weight, mitochondrial function measures remained similar between males and females. Mitochondrial metabolism measures also did not differ by race (*p* > 0.05).

**Table 2:** Descriptive statistics for body composition, thigh composition, and mitochondrial metabolism parameters

	All subjects	Females	Males
	N = 34	n = 19	n = 15
*Body composition and thigh composition*			
Total body weight (kg)	72.3 ± 14.0	67.3 ± 12.1	78.5 ± 14.2*
Fat mass (kg)	20.7 ± 8.9	20.1 (16.7, 24.1)	17.4 (13.0, 23.4)
Percent fat mass (%)	28.0 ± 8.5	30.2 (26.7, 34.1)	22.5 (18.1, 28.0)*
Lean mass (kg)	48.8 ± 8.7	42.9 (40.2, 45.9)	55.5 (52.6, 58.5)**
VAT (kg)	0.36 ± 0.43	0.16 (0.08, 0.25)	0.53 (0.30, 0.80)**
Thigh skeletal muscle (cm^2^)	137.0 ± 26.7	121.8 (113.8, 130.3)	152.7 (138.9, 168.0)**
Thigh SAT (cm^2^)	60.9 ± 30.5	70 (57.6, 85.1)	37.4 (27.5, 50.9)**
Thigh IMAT (cm^2^)	1.2 ± 1.7	0.65 (0.42, 1.01)	0.78 (0.45, 1.36)
*Monocyte mitochondrial metabolism parameters*			
Basal (pmol/min)^§^	93.9 ± 36.5	94.0 (76.8, 115.2)	93.7 (76.9, 114.3)
ATP-linked (pmol/min)	75.1 ± 26.1	73.8 (60.7, 89.7)	76.5 (64.1, 91.3)
Maximal OCR (pmol/min)	222.9 ± 101.4	212.3 (167.6, 268.8)	237.2 (188.6, 298.4)
Reserve capacity (pmol/min)	123.5 ± 70.8	111.6 (82.7, 150.6)	140.4 (106.6, 184.9)
*Muscular mitochondrial metabolism parameters*			
Skeletal muscle τPCr (s)	32.0 ± 12.3	29.5 (24.7, 35.3)	35.5 (28.7, 44)
ATP maximum (mMolal/s)	0.60 ± 0.24	0.65 (0.56, 0.76)	0.54 (0.42, 0.70)
Initial PCr (mMolal)	19.5 ± 2.7	19.5 ± 2.6	19.5 ± 2.8
PCr depletion (%)	35.2 ± 10.1	33.1 ± 8.9	37.9 ± 11.3

Data are presented as mean ± SD or geometric mean (95% confidence interval) for variables that were natural log transformed. *p < 0.05, **p < 0.01 for comparisons between males and females

^§^n = 32 for all subjects and n = 17 for monocyte mitochondrial metabolism parameters in females

VAT, visceral adipose tissue; SAT, subcutaneous adipose tissue; PMAT, perimuscular adipose tissue; IMAT, intermuscular adipose tissue; OCR, oxygen consumption rate; ATP, adenosine triphosphate; PCr, phosphocreatine

### Correlations between body composition and ex vivo monocyte mitochondrial metabolism

**Fig. 1:**
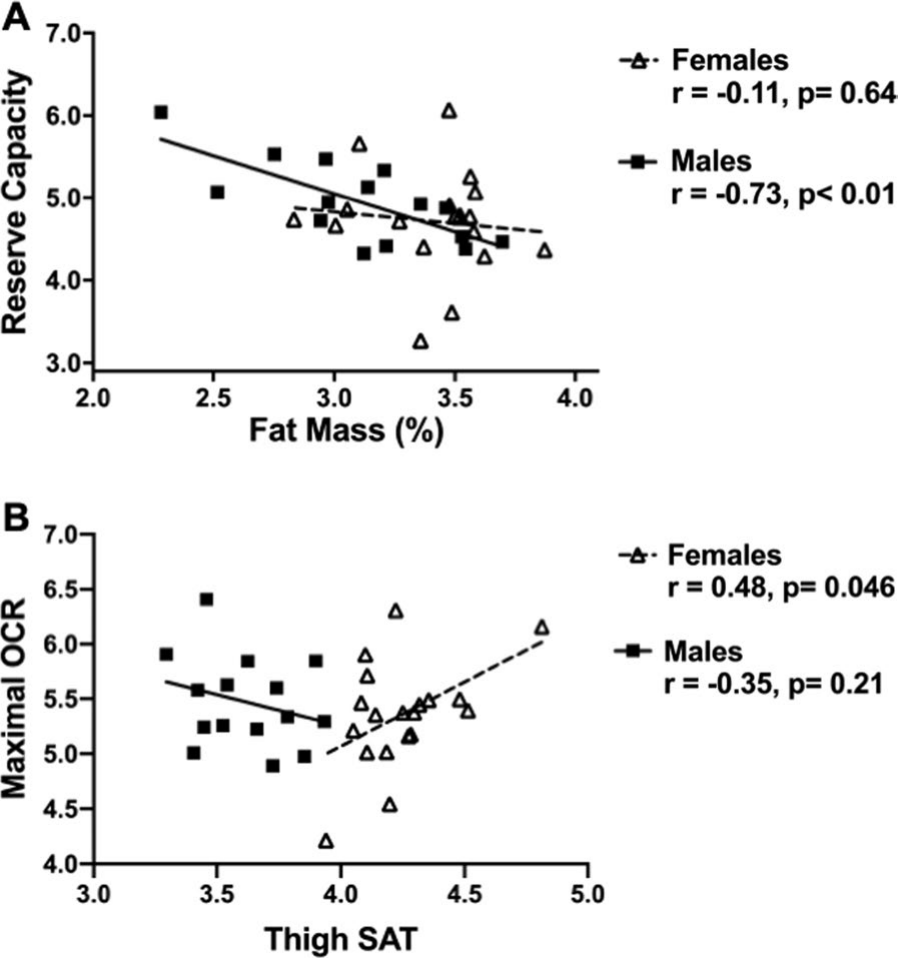
**A** Pearson correlations of the associations between fat mass (%) with reserve capacity for males and females. **B** Partial correlation of the associations between thigh subcutaneous adipose tissue (SAT) with maximal oxygen consumption rate (OCR) for males and females adjusting for fat mass. Partial correlation value between thigh SAT and maximal OCR for all subjects was r = 0.04, *p* = 0.8. All variables were natural log transformed for analyses

Associations of monocyte mitochondrial function and body composition are shown in Table 3. Results were similar for reserve capacity and maximal respiration, so only reserve capacity results are shown with maximal respiration analyses shown in Additional file 1: Table S2. In the full cohort, only fat mass percent was inversely related to reserve capacity (r = − 0.43, *p* = 0.01). There were trends towards significant interactions in the relationships between sex and reserve capacity with body composition measures including fat mass (*p* = 0.08), lean mass (*p* = 0.06), total muscle (*p* = 0.05), thigh SAT (*p* = 0.01). In females, lean mass and thigh muscle were significantly, positively related to monocyte reserve capacity (r = 0.47, *p* = 0.04 and r = 0.47, *p* = 0.04, respectively) and there were no significant correlations with measures of adiposity. In male participants only, fat mass percent (r = − 0.73, *p* = 0.002), fat mass (r = − 0.72, *p* = 0.003), and thigh SAT (r = − 0.75, *p* = 0.001) were inversely related to reserve capacity. The differential relationships between fat mass percent and reserve capacity for males and females are depicted in Fig. 1A. In post-hoc linear regression analyses with additional adjustment for physical activity levels, the relationships between fat mass percent, fat mass, thigh muscle, and thigh SAT with reserve capacity remained (all *p* < 0.05), suggesting these reported relationships are independent of physical activity.

**Table 3:** Associations between monocyte mitochondrial respiration and body composition

	Fat mass (%)	Fat mass (kg)	Lean mass (kg)	VAT (kg)	Thigh muscle (cm^2^)	Thigh SAT (cm^2^)	Thigh IMAT (cm^2^)
All subjects (n = 34)							
Reserve Capacity	− 0.43*	− 0.32	0.32	− 0.14	0.27	− 0.29	− 0.17
Females (n = 19)							
Reserve Capacity	− 0.11	0.06	0.47*	− 0.14	0.47*	0.23	− 0.02
Males (n = 15)							
Reserve Capacity	− 0.73**	− 0.72**	− 0.25	− 0.48	− 0.16	− 0.75**	− 0.49

Bolded values indicate statistical significance

Correlation coefficient is shown. *p < 0.05, ** < 0.01, ^†^*p* = 0.05

Monocyte mitochondrial respiration measures are expressed as pmol of oxygen per minute

VAT, visceral adipose tissue; SAT, subcutaneous adipose tissue; IMAT, intermuscular adipose tissue

In analyses of fat distribution variables adjusting for total fat mass, thigh SAT was positively correlated with maximal OCR (Fig. 1B, r = 0.48, *p* = 0.046) in females only. Among males, the relationship between reserve capacity with thigh SAT was not statistically significant after adjusting for total fat mass (Additional file 1: Table S3).

### Correlations between body composition and in vivo skeletal muscle mitochondrial metabolism

**Fig. 2:**
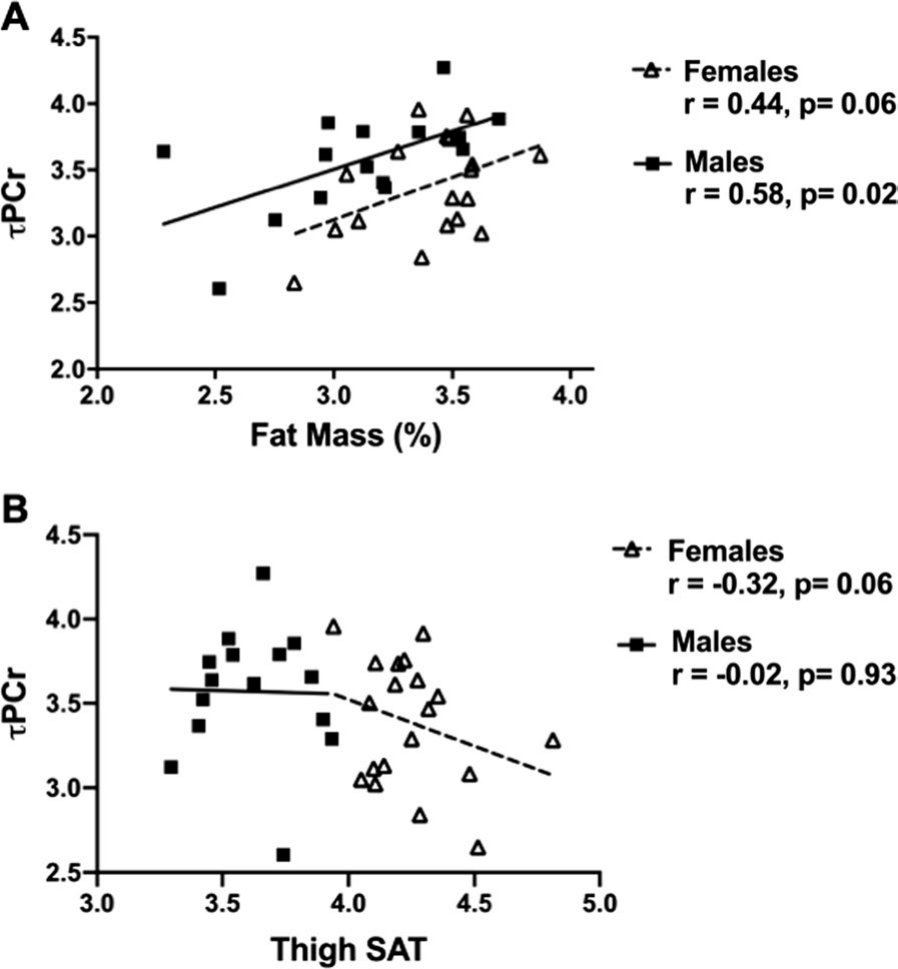
**A** Pearson correlation of the association between fat mass (%) and τPCr for males and females. **B** Partial correlation of the association between thigh subcutaneous adipose tissue (SAT) and τPCr for males and females adjusting for fat mass. Partial correlation value between thigh SAT and τPCr for all subjects was r = − 0.38, *p* = 0.03. All variables were natural log transformed for analyses

Associations between skeletal muscle mitochondrial function and body composition are shown in Table 4. Results were similar for τPCr and ATPmax, so only τPCr findings are presented with ATP max results shown in Additional file 1: Table S2. In all participants, measures of total, visceral, and ectopic adiposity were positively associated with τPCr, including fat mass percent (r = 0.34, *p* = 0.05), fat mass (r = 0.40, *p* = 0.02), VAT (r = 0.41, *p* = 0.02), and thigh IMAT (r = 0.45, *p* = 0.008). There was not evidence of a statistically significant interaction between sex and skeletal muscle mitochondrial function with body composition measures (*p* > 0.05), however, analyses are also presented in a sex stratified manner to mirror the ex vivo monocyte analyses. In females, total adiposity measures were not correlated to τPCr (*p* > 0.05) and only thigh IMAT was positively associated with higher τPCr (r = 0.53, *p* = 0.02). In males, τPCr was significantly associated with total adiposity measures (all *p* < 0.05). Relationships between fat mass percent and τPCr for male and female participants are shown in Fig. 2A. In analyses of fat distribution variables adjusting for total fat mass, relationships between τPCr with VAT and thigh IMAT were no longer statistically significant (Additional file 1: Table S3), although thigh SAT was inversely associated with τPCr (r = − 0.38, *p* = 0.03, Fig. 2B).

**Table 4:** Associations between skeletal muscle mitochondrial metabolism and body composition

	Fat mass (%)	Fat mass (kg)	Lean mass (kg)	VAT (kg)	Thigh muscle (cm^2^)	Thigh SAT (cm^2^)	Thigh IMAT (cm^2^)
All subjects (n = 34)							
τPCr	**0.34**^**†**^	0.40*	0.12	0.41*	0.04	0.14	0.45**
Females (n = 19)							
τPCr	0.44	0.38	− 0.05	0.23	− 0.01	0.19	0.53*
Males (n = 15)							
τPCr	0.58*	0.53*	− 0.16	0.45	− 0.26	0.49	0.33

Bolded values indicate statistical significance

Correlation coefficient is shown. *p < 0.05, **p < 0.01, ^†^*p* = 0.05

VAT, visceral adipose tissue; SAT, subcutaneous adipose tissue; IMAT, intermuscular adipose tissue; ATP, adenosine triphosphate; PCr, phosphocreatine

In post-hoc linear regression analyses with additional adjustment for physical activity levels, the significant relationships between fat mass percent, fat mass, VAT, and thigh IMAT remained in all participants (all *p* < 0.05). In females, relationships of fat mass percent and fat mass with τPCr were slightly enhanced (ß = 0.87 ± 0.37, *p* = 0.03 and 0.53 ± 0.25, *p* = 0.05, respectively), while in males, relationships of fat mass with τPCr and ATP max (ß = 0.41 ± 0.19, *p* = 0.05 and − 0.48 ± 0.22, *p* = 0.05, respectively) were slightly attenuated after adjusting for activity level.

### Correlations between ex vivo monocyte mitochondrial metabolism and in vivo skeletal muscle mitochondrial metabolism with clinical measures

Among comparisons between monocyte and skeletal muscle mitochondrial metabolism with clinical measures, fasting insulin and HOMA-IR were related to a higher τPCr in all participants (r = 0.35, *p* = 0.04 and r = 0.34, *p* = 0.05, respectively). Fasting glucose was not significantly associated with any mitochondrial metabolism measures (all *p *> 0.05).

## Discussion

Despite comparable measures of mitochondrial function between males and females, the associations between mitochondrial metabolism parameters and body composition differed by sex, particularly regarding monocyte metabolism and body composition. In females, measures of total adiposity (fat mass and fat mass percent) were not related to mitochondrial functional impairment, but higher lean mass and muscle area were correlated to greater monocyte mitochondrial respiration. Also, more thigh SAT was related to higher monocyte mitochondrial metabolism in females. Conversely, among male participants, higher total body, visceral, and thigh adiposity were strongly related to impaired monocyte and muscle mitochondrial metabolism. Among clinical measures, higher levels of fasting insulin and HOMA-IR were linked to diminished skeletal muscle metabolism.

In sex-stratified analyses, total adiposity, in males only, was strongly related to impaired monocyte and muscle mitochondrial function. These findings support evidence indicating lower cardiometabolic disease risk in premenopausal females despite higher body fat [30]. Prior research has linked obesity with impaired mitochondrial metabolism [19, 31–33], possibly caused by obesity-related factors such as increased inflammation, greater oxidant production, changes in mitochondrial dynamics, and/or excessive energy intake [10, 14]. Impaired mitochondrial function has important medical implications as it typically precedes onset of metabolic diseases [34]. Use of monocytes as circulating mitochondrial biosensors allows for the assessment of overall mitochondrial health using a relatively non-invasive procedure and provides a unique opportunity to assess the onset and progression of metabolic disease. Here, findings confirm a relationship between increased total body adiposity and impaired mitochondrial function in monocytes and skeletal muscle mitochondrial function, particularly in males. These results suggest increased adiposity may damage monocyte mitochondrial function in males, but females may tolerate greater proportions of fat mass without compromising mitochondrial function.

In addition to total adiposity, VAT and thigh IMAT were associated with reduced skeletal muscle bioenergetics, but these relationships were not independent of total fat mass. Findings regarding IMAT underscore the importance of muscle quality in muscle mitochondrial function. Both VAT and IMAT are recognized as dysfunctional adipose tissue depots that are closely linked to insulin resistance [3, 35], yet limited research has explored the impact of these fat depots on mitochondrial metabolism [15, 16]. In agreement with findings presented here, non-specific measures of abdominal adiposity (increased waist circumference) have been associated with decreased muscle mitochondrial metabolism in men, although these findings were unadjusted for total adiposity [16]. Diminished muscle mitochondrial oxidative phosphorylation is related to greater insulin resistance and likelihood of prediabetes [36]. Accordingly, in our study, participants with higher fasting insulin and HOMA-IR levels exhibited lower skeletal muscle mitochondrial capacity, despite lacking a metabolic disease diagnosis. Larger studies are needed to determine if visceral and ectopic fat correlate with reduced systemic and tissue-specific mitochondrial metabolism.

Lower body fat accumulation, relative to central adiposity, is associated with better metabolic health and lower disease risk [4]. Specific thigh fat depots may have opposing relationships on metabolic health [23, 35]. Findings here corroborate that concept and demonstrate sex differences in the associations between thigh fat depots with mitochondrial function. Greater thigh SAT was associated with better mitochondrial function in females, but worse mitochondrial function in males. In line with this, Goss et al*.* reported higher insulin sensitivity related to thigh SAT, independent of total adiposity, in early post-menopausal females [23]. Another pilot study reported differential, but not statistically significant relationships, between thigh SAT and mitochondrial respiration in elderly, sedentary females compared to males [19]. Conversely, a study of women living with HIV found that participants with the highest total and lower body adiposity exhibited lower monocyte reserve capacity, but these results may reflect the pathophysiology of HIV or impacts of HIV-therapy, and distinction of specific thigh fat depots was not available [21]. In aggregate, data presented herein demonstrate unique relationships between males and females with thigh fat depots and mitochondrial metabolism requiring further investigation.

While gluteal-femoral fat and peripheral fat storage is protective against disease in both sexes, this fat pattern is more common in females [4]. In women, gluteal-femoral fat tissue expansion primarily occurs through adipocyte hyperplasia, possibly preserving mitochondrial function despite excessive energy intake and/or adiposity [37]. In contrast, in men, this fat depot expansion occurs through adipocyte hypertrophy, which leads to hypoxia and metabolic dysfunction [38]. Findings herein support data proposing subcutaneous gluteal-femoral fat storage does not correlate to impaired metabolic functions and, therefore, may protect against metabolic disease in healthy, pre-menopausal females.

Among female participants only, lean mass and muscle area were moderately associated with greater monocyte respiration. Independent of BMI, another report also showed a positive relationship between mitochondrial function and lean mass [19]. In addition to providing structural maintenance, lean mass (predominantly comprised of skeletal muscle) offers important metabolic functions, including energy and protein metabolism [39, 40]. Higher appendicular lean mass was related to lower lipid levels in females, improved insulin sensitivity in males, and lower fibrinogen concentrations in both sexes [30]. Higher lean mass is also related to greater cardiorespiratory fitness [41], which is linked to improved metabolic health, lower disease risk, and better mitochondrial function [32, 42]. Conversely, low lean mass and concomitantly decreased muscle strength (i.e., sarcopenia), has detrimental impacts on clinical outcomes and mortality [39], particularly in women [43]. Treatments promoting the synthesis and maintenance of lean mass may be integral to reverse adverse outcomes of mitochondrial dysfunction [39, 44].

Males and females exhibited similar mitochondrial metabolism parameters but unique relationships with body composition. The mechanisms driving these dissimilar relationships is unclear and requires further investigation. Sex steroids may influence mitochondrial dynamics [45] and modulate fat mass partitioning and function [4]. Estrogen may help protect from VAT and ectopic fat deposition, instead promoting subcutaneous fat storage [46] while protecting skeletal muscle from damage by reducing inflammation and oxidant production [47]. Sex steroids also influence endocrine function of adipose tissue, which impacts whole body metabolism. For example, while estrogen induces leptin secretion, androgens inhibit leptin secretion [4]. Differences may also be attributed to distinctions in lipolytic activity and fuel utilization by sex [48]. Future research investigating the sex differences in relationships between body composition and mitochondrial function will help improve understanding of sexual dimorphisms linking metabolic and endocrine functions to disease.

This study has several strengths. Sex-stratified analyses highlight novel relationships and facilitated interpretations that would go undetected without considering sex as a biological variable. Including two measures of tissue-specific mitochondrial oxidative phosphorylation provided a more robust assessment of mitochondrial metabolism rather than a single measurement. Use of both DXA and MRI enabled gold-standard assessment of total body composition and fat distribution linked to bioenergetics. Standardizing the MRS exercise to each participant’s lean mass helped to reduce between-subject variation arising from different amounts of muscle mass. Further, to our knowledge, this is the first study linking DEXA-derived body composition and monocyte bioenergetics in healthy participants, providing additional evidence for the use of circulating cells as a metabolic biosensor and enabling a move away from invasive tissue biopsies. Limitations of this study included the cross-sectional design of the study, the small sample size, and generalizability to young adults. This pilot study may have been inadequately powered to detect significant differences between multiple groups and account for multiple comparisons and outcomes. While VAT and thigh fat depots were measured, additional ectopic fat depots not assessed, such as hepatic and pancreatic fat, may contribute to metabolic impairments. Finally, physical fitness, which could induce changes in mitochondrial function, was assessed by self-report only. Future longitudinal research in this area should investigate if observed differences in male and females persist with aging, menopausal status, and with disease onset and progression.

### Perspectives and significance

Greater understanding of mechanisms driving sex differences in disease occurrence is needed. This study presents intriguing results wherein differences were observed in the associations between body composition and mitochondrial function for males and females. Moreover, the results demonstrate tissue-specific associations between monocyte and skeletal muscle metabolism with body composition and clinical measures. These preliminary findings may be used to generate hypotheses to test mechanisms that underlie sex differences in disease occurrence.

## Conclusions

The findings of this study demonstrate novel sex-specific differences in the associations between circulating monocytes and skeletal muscle mitochondrial energy metabolism with adipose tissue, adipose tissue distribution, and lean mass. In males only, measures of adiposity were inversely related to mitochondrial function. In marked contrast, in females, total adiposity measures were not associated with impaired mitochondrial functions, while measures of lean mass and thigh SAT were significantly, positively associated with monocyte mitochondrial respiration. Higher levels of insulin and HOMA-IR were also linked to worse skeletal muscle metabolism. Understanding the mechanisms linking these relationships may inform targeted, sex-specific interventions to improve mitochondrial energy metabolism and metabolic health. Larger studies are needed to confirm these results.

## Supplementary Information

**Additional file 1. Supplemental Table 1.** Standard breakfast served prior to in vivo assessment of skeletal muscle mitochondrial function. **Supplemental Table 2.** Pearson correlations between monocyte (maximal OCR) and skeletal muscle (ATP max) mitochondrial respiration and body composition. **Supplemental Table 3.** Partial correlations between monocyte and skeletal muscle mitochondrial metabolism and body composition.

## Data Availability

Data is available upon request and at the discretion of the senior author.

## Abbreviations

ATPmax: Maximal rate of ATP (adenosine triphosphate) synthesis
BMI: Body mass index
FM: Fat mass
IMAT: Intermuscular adipose tissue
NWO: Normal weight obesity
OCR: Oxygen consumption rate
SAT: Subcutaneous adipose tissue
τPCr: Recovery time constant for phosphocreatine
VAT: Visceral adipose tissue

## References

[1] Heymsfield SB, Wadden TA (2017). Mechanisms, Pathophysiology, and Management of Obesity. N Engl J Med.

[2] Okorodudu DO, Jumean MF, Montori VM, Romero-Corral A, Somers VK, Erwin PJ (2010). Diagnostic performance of body mass index to identify obesity as defined by body adiposity: a systematic review and meta-analysis. Int J Obesity (2005).

[3] Neeland IJ, Ross R, Despres JP, Matsuzawa Y, Yamashita S, Shai I (2019). Visceral and ectopic fat, atherosclerosis, and cardiometabolic disease: a position statement. Lancet Diabetes Endocrinol.

[4] Karastergiou K, Smith SR, Greenberg AS, Fried SK (2012). Sex differences in human adipose tissues—the biology of pear shape. Biol Sex Differ.

[5] Ventura-Clapier R, Moulin M, Piquereau J, Lemaire C, Mericskay M, Veksler V (2017). Mitochondria: a central target for sex differences in pathologies. Clin Sci (London, England:1979).

[6] Regitz-Zagrosek V, Oertelt-Prigione S, Prescott E, Franconi F, Gerdts E, Foryst-Ludwig A (2016). Gender in cardiovascular diseases: impact on clinical manifestations, management, and outcomes. Eur Heart J.

[7] Nasrallah CM, Horvath TL (2014). Mitochondrial dynamics in the central regulation of metabolism. Nat Rev Endocrinol.

[8] Willems PH, Rossignol R, Dieteren CE, Murphy MP, Koopman WJ (2015). Redox homeostasis and mitochondrial dynamics. Cell Metab.

[9] Bajpeyi S, Pasarica M, Moro C, Conley K, Jubrias S, Sereda O (2011). Skeletal muscle mitochondrial capacity and insulin resistance in type 2 diabetes. J Clin Endocrinol Metab.

[10] De Mello AH, Costa AB, Engel JDG, Rezin GT (2018). Mitochondrial dysfunction in obesity. Life Sci.

[11] Tyrrell DJ, Bharadwaj MS, Jorgensen MJ, Register TC, Molina AJ (2016). Blood cell respirometry is associated with skeletal and cardiac muscle bioenergetics: implications for a minimally invasive biomarker of mitochondrial health. Redox Biol.

[12] Liu Y, Gu Y, Yu X (2017). Assessing tissue metabolism by phosphorous-31 magnetic resonance spectroscopy and imaging: a methodology review. Quant Imaging Med Surg.

[13] Ferrick DA, Neilson A, Beeson C (2008). Advances in measuring cellular bioenergetics using extracellular flux. Drug Discovery Today.

[14] Bournat JC, Brown CW (2010). Mitochondrial dysfunction in obesity. Curr Opin Endocrinol Diabetes Obes.

[15] Lynam-Lennon N, Connaughton R, Carr E, Mongan AM, O'Farrell NJ, Porter RK (2014). Excess visceral adiposity induces alterations in mitochondrial function and energy metabolism in esophageal adenocarcinoma. BMC Cancer.

[16] Chanseaume E, Barquissau V, Salles J, Aucouturier J, Patrac V, Giraudet C (2010). Muscle mitochondrial oxidative phosphorylation activity, but not content, is altered with abdominal obesity in sedentary men: synergism with changes in insulin sensitivity. J Clin Endocrinol Metab.

[17] O'Brien LC, Wade RC, Segal L, Chen Q, Savas J, Lesnefsky EJ (2017). Mitochondrial mass and activity as a function of body composition in individuals with spinal cord injury. Physiol Rep.

[18] Lalia AZ, Dasari S, Johnson ML, Robinson MM, Konopka AR, Distelmaier K (2016). Predictors of whole-body insulin sensitivity across ages and adiposity in adult humans. J Clin Endocrinol Metab.

[19] Bharadwaj MS, Tyrrell DJ, Leng I, Demons JL, Lyles MF, Carr JJ (2015). Relationships between mitochondrial content and bioenergetics with obesity, body composition and fat distribution in healthy older adults. BMC obesity.

[20] Johannsen DL, Conley KE, Bajpeyi S, Punyanitya M, Gallagher D, Zhang Z (2012). Ectopic lipid accumulation and reduced glucose tolerance in elderly adults are accompanied by altered skeletal muscle mitochondrial activity. J Clin Endocrinol Metab.

[21] Willig AL, Kramer PA, Chacko BK, Darley-Usmar VM, Heath SL, Overton ET (2017). Monocyte bioenergetic function is associated with body composition in virologically suppressed HIV-infected women. Redox Biol.

[22] Chacko BK, Smith MR, Johnson MS, Benavides G, Culp ML, Pilli J (2019). Mitochondria in precision medicine; linking bioenergetics and metabolomics in platelets. Redox Biol.

[23] Goss AM, Gower BA (2012). Insulin sensitivity is associated with thigh adipose tissue distribution in healthy postmenopausal women. Metab Clin Exp.

[24] Stefan DDCF, Andrasescu A, Popa E, Lazariev A, Vescovo E, Strbak O, Williams S, Starcuk Z, Cabanas M, van Ormondt D, Graveron-Demilly D (2009). Quantitation of magnetic resonance spectroscopy signals: the jMRUI software package. Meas Sci Technol.

[25] Vanhamme L, van den Boogaart A, Van Huffel S (1997). Improved method for accurate and efficient quantification of MRS data with use of prior knowledge. J Magn Resonance (San Diego, Calif:1997).

[26] Blei ML, Conley KE, Kushmerick MJ (1993). Separate measures of ATP utilization and recovery in human skeletal muscle. J Physiol.

[27] McCully KK, Fielding RA, Evans WJ, Leigh JS, Posner JD (1993). Relationships between in vivo and in vitro measurements of metabolism in young and old human calf muscles. J Appl Physiol (Bethesda, Md:1985).

[28] Bellissimo MP, Cai Q, Ziegler TR, Liu KH, Tran PH, Vos MB (2019). Plasma high-resolution metabolomics differentiates adults with normal weight obesity from lean individuals. Obesity (Silver Spring, Md).

[29] Group TI. Guidelines for Data Processing and Analysis of the International Physical Activity Questionnaire. 2005. http://www.ipaq.ki.se. 25376692

[30] Schorr M, Dichtel LE, Gerweck AV, Valera RD, Torriani M, Miller KK (2018). Sex differences in body composition and association with cardiometabolic risk. Biol Sex Differ.

[31] Tyrrell DJ, Bharadwaj MS, Van Horn CG, Marsh AP, Nicklas BJ, Molina AJ (2015). Blood-cell bioenergetics are associated with physical function and inflammation in overweight/obese older adults. Exp Gerontol.

[32] Larson-Meyer DE, Newcomer BR, Hunter GR, McLean JE, Hetherington HP, Weinsier RL (2000). Effect of weight reduction, obesity predisposition, and aerobic fitness on skeletal muscle mitochondrial function. Am J Physiol Endocrinol Metab.

[33] Bakkman L, Fernstrom M, Loogna P, Rooyackers O, Brandt L, Lagerros YT (2010). Reduced respiratory capacity in muscle mitochondria of obese subjects. Obes Facts.

[34] Patti ME, Corvera S (2010). The role of mitochondria in the pathogenesis of type 2 diabetes. Endocr Rev.

[35] Boettcher M, Machann J, Stefan N, Thamer C, Haring HU, Claussen CD (2009). Intermuscular adipose tissue (IMAT): association with other adipose tissue compartments and insulin sensitivity. J Magn Reson Imaging.

[36] Jheng HF, Tsai PJ, Guo SM, Kuo LH, Chang CS, Su IJ (2012). Mitochondrial fission contributes to mitochondrial dysfunction and insulin resistance in skeletal muscle. Mol Cell Biol.

[37] Muir LA, Neeley CK, Meyer KA, Baker NA, Brosius AM, Washabaugh AR (2016). Adipose tissue fibrosis, hypertrophy, and hyperplasia: Correlations with diabetes in human obesity. Obesity (Silver Spring, Md).

[38] Tchoukalova YD, Koutsari C, Karpyak MV, Votruba SB, Wendland E, Jensen MD (2008). Subcutaneous adipocyte size and body fat distribution. Am J Clin Nutr.

[39] Deutz NEP, Ashurst I, Ballesteros MD, Bear DE, Cruz-Jentoft AJ, Genton L (2019). The underappreciated role of low muscle mass in the management of malnutrition. J Am Med Dir Assoc.

[40] Argiles JM, Campos N, Lopez-Pedrosa JM, Rueda R, Rodriguez-Manas L (2016). Skeletal muscle regulates metabolism via interorgan crosstalk: roles in health and disease. J Am Med Dir Assoc.

[41] Carbone S, Popovic D, Lavie CJ, Arena R. Obesity, body composition and cardiorespiratory fitness in heart failure with preserved ejection fraction. Future Cardiol. 2017. 10.2217/fca-2017-0023 28795590

[42] Kodama S, Saito K, Tanaka S, Maki M, Yachi Y, Asumi M (2009). Cardiorespiratory fitness as a quantitative predictor of all-cause mortality and cardiovascular events in healthy men and women: a meta-analysis. JAMA.

[43] Batsis JA, Mackenzie TA, Barre LK, Lopez-Jimenez F, Bartels SJ (2014). Sarcopenia, sarcopenic obesity and mortality in older adults: results from the National Health and Nutrition Examination Survey III. Eur J Clin Nutr.

[44] Coen PM, Musci RV, Hinkley JM, Miller BF (2018). Mitochondria as a target for mitigating sarcopenia. Front Physiol.

[45] Lynch S, Boyett JE, Smith MR, Giordano-Mooga S (2020). Sex hormone regulation of proteins modulating mitochondrial metabolism, dynamics and inter-organellar cross talk in cardiovascular disease. Front Cell Dev Biol.

[46] Tramunt B, Smati S, Grandgeorge N, Lenfant F, Arnal JF, Montagner A, et al. Sex differences in metabolic regulation and diabetes susceptibility. Diabetologia. 2019. 10.1007/s00125-019-05040-3 PMC6997275 31754750

[47] Ikeda K, Horie-Inoue K, Inoue S (2019). Functions of estrogen and estrogen receptor signaling on skeletal muscle. J Steroid Biochem Mol Biol.

[48] Varlamov O, Bethea CL, Roberts CT (2014). Sex-specific differences in lipid and glucose metabolism. Front Endocrinol (Lausanne).

