# Supplementary material for: Sex differences in the relationships between body composition, fat distribution, and mitochondrial energy metabolism: a pilot study
Source: Nutr Metab (Lond). 2022 May 21;19:37. doi: 10.1186/s12986-022-00670-8 (PMC9123728; doi:10.1186/s12986-022-00670-8)
Supplement: Supplementary file 1 — Additional file 1. Supplemental Table 1. Standard breakfast served prior to in vivo assessment of skeletal muscle mitochondrial function. Supplemental Table 2. Pearson correlations between monocyte (maximal OCR) and skeletal muscle (ATP max) mitochondrial respiration and body composition. Supplemental Table 3. Partial correlations between monocyte and skeletal muscle mitochondrial metabolism and body composition. [file 12986_2022_670_MOESM1_ESM.docx]

| **Supplemental Table 1**. Standard breakfast served prior to in vivo assessment of skeletal muscle mitochondrial function | | | | |
| --- | --- | --- | --- | --- |
| **Breakfast Item** | **Energy (kcal)** | **Fat**  **(gm)** | **Carbohydrate (gm)** | **Protein**  **(gm)** |
|  |  |  |  |  |
| Honey Nut Cheerios, 1 oz | 111 | 1.5 | 22.3 | 2.0 |
| 1% milk, 1 cup | 102 | 2.4 | 12.2 | 8.2 |
| Yoplait Light Yogurt, 6 oz. | 100 | 0.19 | 19.9 | 5.2 |
| Banana, medium | 105 | 0.39 | 27.0 | 1.3 |
| Blueberry muffin | 337 | 12.5 | 49.7 | 6.8 |
| Total for Meal | 756 | 17.0 | 131.1 | 23.5 |
| % kcal | - | 20% | 68% | 12% |

Water was provided ad libitum to all participants.

| **Supplemental Table 2.** Pearson correlations between monocyte (maximal OCR) and skeletal muscle (ATP max) mitochondrial respiration and body composition | | | | | | | |
| --- | --- | --- | --- | --- | --- | --- | --- |
|  | **Fat mass (%)** | **Fat mass (kg)** | **Lean mass (kg)** | **VAT**  **(kg)** | **Thigh muscle (cm^2^)** | **Thigh SAT**  **(cm^2^)** | **Thigh IMAT (cm^2^)** |
| **All subjects (n=34)** |  |  |  |  |  |  |  |
| Maximal OCR | **-0.36*** | -0.26 | 0.23 | -0.18 | 0.17 | -0.20 | -0.15 |
| ATP max | **-0.34^†^** | **-0.39*** | -0.14 | **-0.42*** | -0.03 | -0.17 | **-0.37*** |
| **Females (n=19)** |  |  |  |  |  |  |  |
| Maximal OCR | -0.04 | 0.10 | 0.38 | -0.18 | 0.33 | 0.32 | -0.03 |
| ATP max | -0.31 | -0.29 | -0.03 | -0.11 | 0.01 | -0.21 | -0.41 |
| **Males (n=15)** |  |  |  |  |  |  |  |
| Maximal OCR | **-0.67**** | **-0.64**** | -0.17 | -0.42 | -0.13 | **-0.70**** | -0.36 |
| ATP max | **-0.60*** | **-0.55*** | 0.14 | -0.48 | 0.23 | -0.49 | -0.33 |
| Correlation value is shown. *p< 0.05, **<0.01, ^†^p=0.05.  Monocyte mitochondrial respiration measures is expressed as pmol of oxygen per minute. Skeletal muscle mitochondrial metabolism measure is expressed as mMolal/s (ATP max)  Abbreviations: OCR, oxygen consumption rate; VAT, visceral adipose tissue; SAT, subcutaneous adipose tissue; IMAT, intermuscular adipose tissue | | | | | | | |

| **Supplemental Table 3.** Partial correlations between monocyte and skeletal muscle mitochondrial metabolism and body composition | | | |
| --- | --- | --- | --- |
|  | **VAT**  **(kg)** | **Thigh SAT**  **(cm^2^)** | **Thigh IMAT (cm^2^)** |
| **All subjects (n=34)** |  |  |  |
| Reserve Capacity | 0.07 | -0.05 | 0.008 |
| τPCr | 0.23 | **-0.38*** | 0.27 |
| **Females (n=19)** |  |  |  |
| Reserve Capacity | -0.30 | 0.37 | -0.05 |
| τPCr | -0.12 | -0.32 | 0.42 |
| **Males (n=15)** |  |  |  |
| Reserve Capacity | 0.37 | -0.33 | 0.40 |
| τPCr | 0.0003 | -0.02 | -0.24 |
| Partial correlation value is shown. *p< 0.05, **<0.01, ^†^p=0.05. All partial correlations are adjusted for fat mass. Partial correlations with Thigh IMAT are also adjusted for thigh muscle area. Monocyte mitochondrial respiration measures are expressed as pmol of oxygen per minute. Skeletal muscle mitochondrial metabolism measures are expressed as seconds (τPCr)  Abbreviations: OCR, oxygen consumption rate; VAT, visceral adipose tissue; SAT, subcutaneous adipose tissue; IMAT, intermuscular adipose tissue | | | |
